# Case report: Successful multimodal assessment and management of chemothorax

**DOI:** 10.3389/fsurg.2022.921968

**Published:** 2022-07-26

**Authors:** Teodora Panza, Rosatea Quercia, Francesca Signore, Giulia De Iaco, Debora Brascia, Doroty Sampietro, Anna Rita Gasbarro, Maria Dell’Aera, Vito Lorusso, Giuseppe Marulli, Angela De Palma

**Affiliations:** ^1^ Unit of Thoracic Surgery, Department of Emergency and Organ Transplantation, University of Bari “Aldo Moro,” Bari, Italy; ^2^ Pharmacy Unit, University Hospital Polyclinic of Bari, Bari, Italy; ^3^ Medical Oncology Unit, IRCCS Istituto Tumori “Giovanni Paolo II,” Bari, Italy

**Keywords:** Port-A-Cath, pleural effusion, chemothorax, chemotherapy, dislocation, thoracoscopy, video-assisted thoracic surgery, multidisciplinary management

## Abstract

Dislocation or wrong placement of central venous catheters into the pleural cavity is rare, but if undiagnosed, may cause major, sometimes life-threatening, complications (pneumothorax, hemothorax, infection, and migration) and accidental pleural effusion due to intravenous injection of fluids containing drugs (i.e. chemotherapy, antibiotics, parenteral nutrition, other). We report a rare case of pleural effusion consisting of chemotherapy (chemothorax) directly injected into the pleural cavity due to the wrong placement of a central venous catheter (Porth-A-Cath) in a woman with breast cancer. A multidisciplinary management consisting of antidote administration, followed by removal of the venous device and washing of the pleural cavity through video-assisted thoracic surgery (VATS), avoided any major complication related to the adverse event.

## Introduction

Pleural effusion is a common entity in clinical practice with varying etiological origin. Therefore, treatment and prognosis largely depend on its cause. Thoracoscopic approach by video-assisted thoracic surgery (VATS) has been established to be the most sensitive and accurate method in the diagnosis and treatment of pleural effusion over the years (1–3).

Dislocation or wrong placement of central venous catheters into the pleural cavity is rare, but if undiagnosed, may cause major, sometimes life-threatening, complications (pneumothorax, bleeding, infection, migration, and venous air embolism) and accidental pleural effusion due to intravenous injection of fluids containing drugs (i.e. chemotherapy, antibiotics, parenteral nutrition, other) (4).

We report a rare case of pleural effusion consisting of chemotherapy (chemothorax) directly injected into the pleural cavity due to the wrong placement of a central venous catheter (Porth-A-Cath) in a woman with breast cancer, successfully managed by a multidisciplinary approach, consisting of antidote administration, followed by removal of the venous device, and washing of the pleural cavity through VATS.

## Case description

A 54-year-old Caucasian woman affected by breast carcinoma underwent insertion of a central venous catheter (Port-a-Cath) in the right subclavian vein to receive induction chemotherapy at another hospital. The patient had received a biopsy diagnosis of breast carcinoma 3 weeks earlier, requiring, for tumor stage, neo-adjuvant chemotherapy before undergoing demolitive breast surgery. The venous catheter was placed without complications, so no post-procedure chest x-ray was performed to check its correct position. Previous chest x-ray at the time of breast biopsy did not show any pleural effusion.

The patient received first chemotherapy infusion with Cyclophosphamide and Epirubicin hydrochloride, 2 weeks after catheter placement. A few hours later, she presented to the emergency room of our hospital for right chest pain radiating to the right hypochondrium.

The patient had no fever, a pulse rate of 94 beats per minute, a blood pressure of 150/90 mmHg, a respiratory rate of 22 breaths per minute, and an oxygen saturation of 97% on room air; auscultation of the chest revealed the absence of breath sounds in the right basal hemithorax.

**Figure 1:**
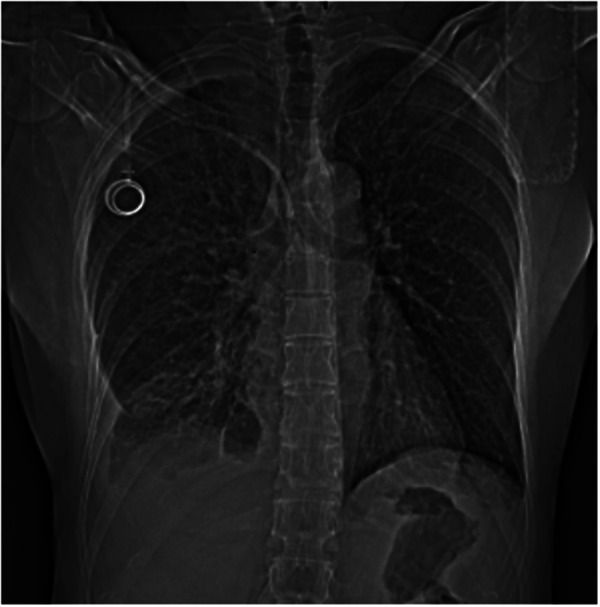
Chest x-ray showing a large right pleural effusion with abnormal position of the distal tip of the Port-a-Cath.

**Figure 2:**
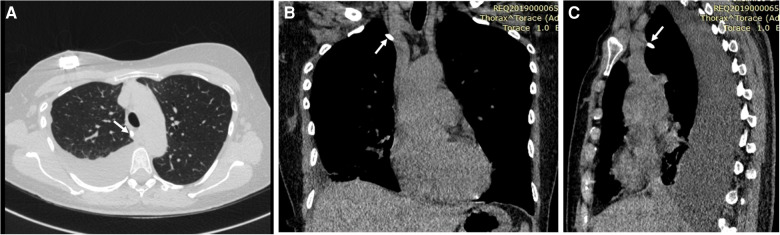
Axial (**A**), coronal (**B**), and sagittal (**C**) chest CT scans showing a large right pleural effusion, complete atelectasis of the lower lobe of the right lung, the proximal tip of the central venous catheter placed tangentially to the right subclavian vein, and the catheter (white arrows) located outside the superior vena cava, reaching with the distal tip the middle mediastinum at the level of the body of D6.

Chest x-ray showed a right pleural effusion with suspected abnormal position of the distal tip of the Port-a-Cath (Figure 1). Thus, we performed chest CT, which allowed to demonstrate the wrong placement of the proximal tip of the catheter, placed tangentially to the right subclavian vein, while the catheter was located outside the superior vena cava, reaching with the distal tip the middle mediastinum at the level of the body of D6; a large right pleural effusion and complete atelectasis of the lower lobe of the right lung were also present (Figure 2).

The patient underwent placement of a right pleural drainage (16 Ch) with removal of serous and blood-tinged pleural effusion, a part of which was sent to the laboratory for cytological and toxicological examination. For the latter, the fluid was analyzed with the gas chromatography–mass spectrometry (GC/MS) recording the presence of Cyclophosphamide and Lidocaine (this last one used during local anesthesia for the placement of the chest tube). The presence of Epirubicin in the pleural fluid could not be demonstrated due to the unavailability of the reactive agent for its detection in our laboratory. Cytological examination of the fluid resulted negative.

Following the placement of the drainage and the total evacuation of the pleural fluid, an antidote (dexrazoxane at the dose of 1,500 mg in 500 ml dilution on the first and second days and 750 mg in 500 ml dilution on the third day) was intravenously administered to the patient.

**Figure 3:**
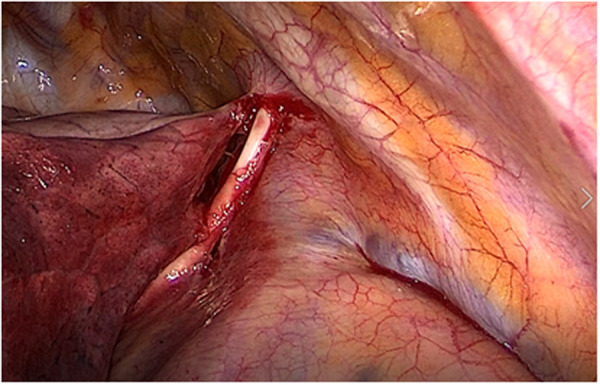
Surgical removal of the Port-a-Cath by triportal video-assisted thoracic surgery (VATS): visualization of the central venous catheter located in the pleural cavity, anteriorly to the right subclavian vessels, and running along the superior vena cava.

**Figure 4:**
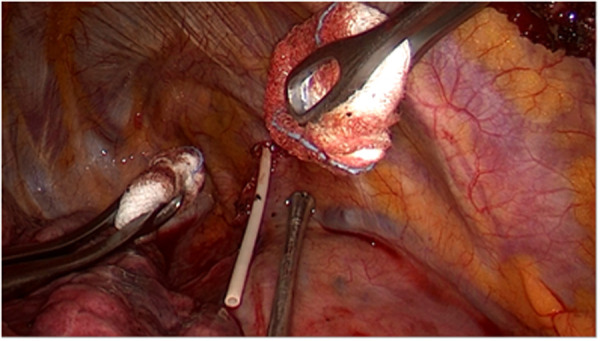
Surgical removal of the Port-a-Cath by triportal VATS: the central venous catheter of the Port-a-Cath is slowly removed, without bleeding, under safe guidance of the thoracoscopic view.

After the first administration of the antidote, the patient underwent surgical removal of the Port-a-Cath by triportal VATS under general anesthesia: this procedure enabled the visualization of a diffuse parietal pleura hyperemia and of the central venous catheter located in the pleural cavity, anteriorly to the right subclavian vessels, and which ran along the superior vena cava (Figure 3). Subsequently, the subcutaneous reservoir was surgically retrieved at the level of the right pectoral region and the central venous catheter of the Port-a-Cath was slowly removed, without bleeding, under safe guidance of the thoracoscopic view (Figure 4). Then, we washed the pleural cavity with 6,000 ml of heated saline solution in order to remove any residual drugs. Finally, a 28 Ch chest tube was placed to drain any post-operative fluids.

On the first post-operative day, a serum increase of pancreatic enzymes (amylase 263 U/L, lipase 3,374 U/L) without symptoms and with ultrasound evidence of slight pancreatic inflammation and leucopenia (1.11 × 10^3^/µl) was detected, probably due to anesthetic drugs; thus, the patient underwent specific medical therapy with gabexate mesilate.

A further pleural fluid sample was analyzed twice (on the second and fifth post-operative day) recording the absence of Cyclophosphamide in both samples.

The daily fluid output from the chest tube was as follows: 350, 720, 360, 150, 300, 220, 100, and 80 ml, thus with a total fluid output of 2,280 ml. On the eighth post-operative day, the chest tube was removed, and the patient was discharged the day after, with normalization of the serum values of leukocytes and of pancreatic enzymes. Chest x-ray performed 2 weeks after discharge showed the recurrence of moderate pleural effusion, which was treated with a small-bore pleural drainage (Drentech UNICO^™^), with a total fluid output of 1,150 ml. No further relapse was then observed in the following controls.

## Discussion

Pleural effusion is commonly associated with congestive heart failure, cancer, pneumonia, and pulmonary embolism. Cases of pleural effusion caused by the infusion of chemotherapy into the pleural cavity due to dislocation/misplacement of central venous catheter are exceptional and rarely reported in the literature (4).

Central venous accesses are widely used in hematological/oncological patients for the management of total parenteral nutrition, chemotherapy, or for obtaining blood samples, significantly improving the patient's quality of life. In particular, the Port-A-Cath is a small reservoir connected to a central venous catheter and located in the subcutaneous tissue. Usually, post-implantation chest x-ray is recommended to check the correct position of the venous catheter; however this was not performed in the described patient, likely due to the absence of complications during the procedure.

Most common problems related to Port-A-Cath insertion/placement include thrombotic and infectious complications (5). Catheter migration, dislocation, or misplacement are relatively rare but life-threatening complications with a reported incidence of approximately 0.9%–2% (6). Particularly, the extravasation of cancer drugs (DNA-binding vesicant drugs such as anthracyclines, e.g. Epirubicin) may lead to scar damage at injury sites with immediate symptoms (7, 8). Sometimes, symptoms can appear after several days or weeks (8).

Therefore, in our case, the first priority was to secure the patient, removing the chemotherapeutic agent by placing a pleural drainage. Moreover, after a multidisciplinary discussion with the oncologists who were treating the patient, in order to plan the therapeutic strategy, we immediately began therapy with the antidote to reduce the complications of drug intoxication (7, 9, 10). Indeed, dexrazoxane prevents the potential effects of anthracycline extravasation and is well tolerated by patients (11).

Pronchik and Sexton described a similar case of accidental injection of chemotherapy into the chest treated merely with evacuative thoracentesis; in addition, the Port-a-Cath was not removed after assessing its integrity and patency with a dye study (12). Likewise, Kelly et al. described the case of a patient carrying a Port-a-Cath in an extravascular position resulting in accidental injection of chemotherapy in the pleural cavity, treated with thoracentesis and subsequently with the placement of a pig-tail catheter (4); the Port-a-Cath was removed and repositioned in order to perform the oncologic treatment. In both cases, no multidisciplinary approach was carried out by medical therapy including the antidote. Furthermore, in the case described by Pronchik and Sexton, the lack of removal of the Port-a-Cath could explain the subsequent hospitalization of the patient for fever and neutropenia and the microbiological positivity on blood cultures (12).

In our case, the thoracoscopic approach allowed to confirm the position of the tip of the catheter and its relationship with the mediastinal vessels and to safely remove it. Moreover, VATS enabled us to perform lavage and irrigation of the pleural cavity with a huge amount of saline solution that would not be possible through pleural drainage. The amount of 6,000 ml of heated saline solution to wash the pleural cavity was chosen considering the double of maximal volume of fluid that could be on average contained in a woman's pleural cavity and also due to the fact that this is the maximal amount contained in a sterile saline solution bag available for intraoperative use at our hospital. The procedure was performed in the absence of complications.

The patient presented a recurrence of moderate pleural effusion, treated with a small-bore pleural drainage. On the other hand, Uges et al. described a pleural effusion due to the malposition of a Port-a-Cath treated merely by drainage; the authors did not remove the device, thus probably promoting the development of an empyema, which required subsequent surgical decortication of the lung (8): this complication may also have been favored by the visual limitation of insertion that characterizes simple percutaneous pleural drainage. For this reason, we preferred a VATS approach, which allowed safe removal of the catheter and accurate lavage of the pleural cavity to reduce the toxicity of drugs, as reported by Aguirre et al. (13).

In conclusion, our experience demonstrated the importance of a multidisciplinary approach, combining early medical and surgical treatment in case of accidental pleural extravasation of chemotherapy drugs: in such emergency condition, the VATS approach allows not only to safely remove the malpositioned central venous catheter, but also to assess eventual tissue damage and adequately perform drainage, lavage, and irrigation of the pleural cavity. Moreover, the association of medical therapy with the well-tolerated antidote, dexrazoxane, is recommended to prevent short- and long-term complications due to the toxicity of chemotherapy.

## Data Availability

The raw data supporting the conclusions of this article will be made available by the authors, without undue reservation.
